# Mushroom Poisoning and Acute Liver Injury: A Case-Based Review

**DOI:** 10.7759/cureus.75706

**Published:** 2024-12-14

**Authors:** Sayak Roy, Huzaifa Saleem

**Affiliations:** 1 Acute Medicine, Princess Royal University Hospital, King's College Hospital NHS Foundation Trust, Orpington, GBR

**Keywords:** acute liver injury, amatoxin, benzylpenicillin, mushroom poisoning, n-acetylcysteine (nac)

## Abstract

Mushrooms have always found their way into our dining plates due to their exotic looks and edibility. It is also one of the food items that can lead to fatal hepatotoxicity if the wrong species is picked up. Mushroom poisoning is frequently seen in forest adventure seekers and presents with variable time frames, mainly with acute gastrointestinal symptoms.

Here we discuss a case of mushroom poisoning in a lady in her early sixties who presented with acute liver injury after 6 hours of wild toxic mushroom intake, having severely raised INR (International Normalized Ratio). She was managed with N-acetylcysteine (NAC), vitamin K, and regular benzylpenicillin in the liver-intensive therapy unit (ITU). The data regarding the treatment of mushroom poisoning is not tested in conventional rigorous randomized control trials. However, the role of good supportive care in liver-ITU, the addition of NAC, and benzylpenicillin in some cases, is reassuring. And if that fails, then liver transplant is a viable option.

Her case underscores the critical importance of early diagnosis and immediate initiation of supportive care, including the addition of NAC and regular benzylpenicillin in selected cases. For those not responding to these conventional therapies, the possibility of a liver transplant, as a last resort, must be considered.

## Introduction

Mushroom poisoning poses a significant health hazard as it is a very important delicacy in many countries and contributes to the increase in toxin-induced disease [1]. Most of the poisonings and fatalities are due to amatoxin-containing mushrooms, which are primarily hepatotoxic [2]. Poisoning due to amatoxin-containing mushrooms is on the rise as young adults often mistake them for hallucinogenic ones, and ingestion of poisonous mushrooms in the wilderness is a common mistake by adventure seekers [3]. The Amanita species have many highly toxic mushrooms, including Amanita phalloides, Amanita bisporigera, Amanita brunnescens, Amanita ocreata, Amanita verna, and Amanita virosa. These collections are often called death or destroying angels [4]. Amatoxin is mainly derived from three different species of mushrooms: Amanita, Lepiota, and Galerina [5]. Cooking does not destroy the toxic element of amanitin, and the lethal dose is minimal - 7mg in adult people of 70 kg weight, and this is present in a single piece of that mushroom [6]. Here, we discuss a case of mushroom poisoning with delayed presentation, leading to acute liver injury, and how she was managed.

## Case presentation

A 63-year-old female was brought to the ED (emergency department) after nausea and vomiting. One day before admission, she ingested one small white wild mushroom with a greenish cap while walking in the woods area around 10 AM. She started having symptoms of abdominal pain and nausea almost 8 hours post-ingestion. Then, she presented to the hospital with acute abdominal pain and vomiting, having attempted to manage her symptoms at home. She was brought to the ED by her daughter, whose prompt action was crucial following increasing drowsiness.

On admission, her vitals were BP 108/64, respiratory rate 16, heart rate 63, and temperature 95.9 degrees Fahrenheit. On examination, the patient showed signs of hypovolemia, with dry mucous membranes and a three-second capillary refill time. The abdomen revealed tenderness in the right upper quadrant without guarding or distension. There were no other systemic findings.

On investigations, the laboratory results at admission showed an average complete blood count with a white blood cell (WBC) count of 9.6 x 10^9^/L, with no evidence of acute infection. The coagulation studies, however, were grossly abnormal, with an INR of 3.9 and prothrombin time (PT) of 38.9 seconds, suggesting high bleeding risk. Liver function tests indicated acute liver injury (ALI), with an alanine aminotransferase (ALT) of 5,582 U/L. There was an ongoing acute kidney injury (AKI) with a rise in urea levels from 7.0 in January to 15.7 mmol/L and a rise in creatinine level from 55 µmol/L in September to 122 µmol/L.

It was an acute reaction after only a few hours of taking the mushroom, which was clear from her history. She had complete orientation, and her history was reliable. All her blood for acute hepatitis profile, including viral, auto-immune, serum copper, ceruloplasmin, and complements, was meticulously tested and found to be negative. Her viral gastrointestinal panel done for her nausea and vomiting also came back negative. Her PMH was insignificant for any medicines contributing to this acute presentation.

After discussion with the National Poisons Information Service (NPIS), one of the most important prognostic factors was established to be the period of ingestion to symptom onset, and a duration of fewer than 8 hours is associated with a poor outcome [7]. The patient was then started on N-acetylcysteine (NAC), a medication that acts as a precursor to glutathione, a potent antioxidant in the body, and vitamin K, 10 mg since there are no specific antidotes for mushroom poisoning. An urgent triple-phase computed tomography (CT) scan demonstrated diffuse hepatic steatosis with no evidence of Budd-Chiari syndrome. Differentials in her case were acute hepatitis, Budd-Chiari syndrome, viral gastroenteritis, and toxin-related liver injury. Detailed history, no other etiologies to suggest any other differentials, and a normal liver scan with normal acute hepatitis screen tests helped us to bring down our diagnosis to mushroom ingestion-related acute liver injury.

Despite her initial treatment, her condition continued to deteriorate during her hospitalization. Her coagulation parameters worsened, with the prothrombin time (PT) increasing to 59.4 seconds and the international normalized ratio (INR) rising to 6.1, which raised significant concerns about potential bleeding complications. The activated partial thromboplastin time (aPTT) was also prolonged to 46.4 seconds, and aspartate aminotransferase (AST) levels soared to 5,468 U/L, confirming acute liver injury (ALI). Given the alarming changes in her blood results, she was transferred to the liver intensive therapy unit (ITU), where she received regular doses of benzylpenicillin for five days, along with N-acetylcysteine (NAC) and vitamin K. NAC was given as 6.25 mg/kg/hour continuous infusion until INR <1.5 or 5 days, and Benzylpenicillin was given at a 2.4 gm IV 4 hourly.

Over the following days, renal function improved to 4.9 mmol/L, and creatinine returned to normal at 49 µmol/L by day 5. Liver enzyme levels showed improvement, with AST dropping to 1,715 U/L on day 5. By day 6, there was a gradual improvement in coagulation parameters, with PT falling to 18.6 s and INR improving to 1.8. This positive trend continued and stabilized at 15.8 sec and 1.5 by day 7. The patient's liver enzyme levels showed improvement, with AST falling to 232 U/L on day 7 and 83 U/L by day 9. By this time, the patient had significant symptomatic relief and was in a stable hemodynamic condition. This stability allowed for her discharge with appropriate follow-up advice. A summary of changes in her coagulation profile over time is given in Table 1.

**Table 1:** Summary of changes in coagulation profile over time PT, Prothrombin time; INR, International normalized ratio; APTT, activated partial thromboplastin time

Component (Latest Ref. Range)	28/9	29/9	30/9	1/10	2/10	3/10	4/10	5/10	15/10
PT (9.7-12.0 sec)	38.9-54.4 in 12 hours	45.6	21.2	18.6	15.8	13.4			
INR (0.8-1.2 ratio)	3.9-6.1 in 12 hours		2.3			1.2	1.2	1.1	0.9
APTT (20.0-29.0 sec)	55.4	58.4	38.4	36.5	32.1	30.9			
Clauss Fibrinogen (1.70-4.90 g/L)	1.54		1.37	1.60	1.82				

Changes in liver function tests over time are presented in Table 2.

**Table 2:** Changes in liver function tests over time AST, aspartate aminotransferase; ALT, alanine aminotransferase; GGT, gamma-glutamyl transferase

Component Latest Ref Range	27/9	28/9	29/9	30/9	1/10	2/10	3/10	4/10	5/10	15/10
ALT 10-35 U/L	5,582	6,651					1,388			103
AST 10-35 U/L		5695	5468	860	464	232	134	97	83	
Albumin 35-50 g/L	44	34	30	29	29	29	28	27	28	35
Alkaline Phosphatase (ALP) 30-130 U/L	167	151	150	156	168	165	208	237	265	159
GGT 1-55 U/L	140	122	155	263	297	313	456	571	700	253
Total Bilirubin <21 µmol/L	62	74	70	133	135	130	123	112	87	24
Total Protein 60-80 g/L	66	48	43	42	41	45	47	46	51	64
Globulin 20-35 g/L	22	14	13	13	13	16	19	19	23	29

Changes in her renal function tests, along with changes in sodium and potassium are summarized in Table 3below.

**Table 3:** Summary of changes in renal function tests, sodium and potassium over time eGFR, estimated glomerular filtration rate

Component (Latest Ref Range)	27/9	28/9	29/9	30/9	1/10	2/10	3/10	4/10	5/10	15/10
Creatinine (47-99 µmol/L)	122	60	49	42	36	46	51	50	58	64
Sodium (135-145 mmol/L)	134	138	140	139	138	136	134 (L)	136	134	138
Potassium (3.5-5.3 mmol/L)	5.3	4.8	3.9	4.8	4.2	4.3	4.2	3.9	4.7	4.6
eGFR by CKD-EPI (2009) NA mL/min/1.73m^2^	41	>90	>90	>90	>90	>90	>90	>90	>90	89
Urea (2.5-7.8 mmol/L)	15.7	8.2	4.9	1.9	1.7	2.2	3.9	3.3	3.1	5.6

## Discussion

The only definitive treatment for worsening acute liver failure due to amatoxin poisoning is liver transplantation [8]. Other treatments are mostly supportive, but the major drawback to those therapies is that randomized controlled trials do not rigorously verify them [8]. Patients who often present late with gastrointestinal toxicity and often get better temporarily before proceeding to fulminant liver failure should always be transferred to centers equipped to perform an emergency liver transplantation. Prognosis is better in patients presenting with amatoxin-induced acute liver injury not progressing to acute liver failure than those with acute liver failure on presentation [5]. Acute liver failure (ALF) is a lethal, potentially reversible condition where hepatic function deteriorates rapidly, as evidenced by deranged coagulation (INR >1.5), jaundice, and development of hepatic encephalopathy where there was no prior evidence of liver disease [9]. Amatoxin inside the mushrooms determines liver failure as it causes liver necrosis. The necrosis is due to the cellular damage caused by problems in fragmentation and segregation of all nuclear components [10]. After binding, the amatoxins inhibit ribonucleic acid (RNA) polymerase II, without which there is a rapid decline in messenger RNA levels, leading to a decrease in protein synthesis and hepatocyte destruction [11]; studies, both toxicological and experimental, point to the central role of inflammation in these mushroom-induced hepatotoxicities. Tumor necrosis factor alpha (TNF-α) has been shown in In vivo studies to enhance amatoxin actions and increase hepatotoxicity, and pre-treatment with anti-TNF antibodies leads to a lesser degree of liver injury. Inflammation plays a central role in mushroom-induced hepatotoxicity, with TNF-α enhancing amatoxin actions and increasing hepatotoxicity. Pre-treatment with anti-TNF antibodies has been shown to reduce the degree of liver injury [11]. Superoxide dismutase activity and reactive oxygen species (ROS) generation are increased by α-amanitin (cyclic toxic peptide in mushroom A. phalloides) [11].

Classic symptoms in order of presentation are nausea and vomiting, which accounts for 95.8%, diarrhea for 62.5%, and abdominal pain, which is seen in 50%. Various reasons are attributable to the abdominal pain that includes congestion of the liver, liver capsule stretching, as well as gastroenteritis might also contribute. Clinical courses are variable from the time of ingestion to the full symptom development [12]. Myocarditis has also been reported due to mushroom poisoning [13]. There have been reports of cholinergic toxidrome that presents with acute gastrointestinal symptoms - starting with epigastric pain, followed by vomiting and diarrhea, finally terminating in miosis, palpitations, and diaphoresis [14].

Prognosis is often calculated based on the latency time of symptom onset from the time of congestion - patients run a milder course if symptom onset is < 6 hours, and multi-organ damage with fatalities is associated with those having latency > 6 hours [15]. A retrospective case-control study involving 52 patients showed that INR and the European Foundation for the Study of Chronic Liver Failure-organ failure (CLIF-C OF) score can be used to evaluate the prognosis of patients with amanita phalloides poisoning [16]. ALI patients do better prognostically than those having ALF [17]. According to the European Association for the Study of the Liver (EASL) manual, the definition of ALI refers to an elevated serum transaminase level, presence of moderately severe coagulopathy (INR > 1.5), symptoms acute in onset on a background of absence of cirrhosis and hepatic encephalopathy (HE); ALF definition requires the presence of any degree of HE plus other features of ALI [18].

Therapies for Amanita phalloides poisoning, as suggested in a literature review done by Ye and Liu, include - gastric decontamination using activated charcoal with gastric lavage within 1 hour of ingestion, enhancing amatoxin elimination by diuresis and biliary drainage, supportive therapies combined with plasmapheresis, use of molecular absorbent regenerating system (MARS), and fractionated plasma separation and adsorption (FPSA) within 36-48 hours of ingestion [10]. The review also suggested roles of Benzylpenicillin, N-acetylcysteine, and silymarin, with emergency liver transplantation being the final gold standard approach to fulminant ALF not responding to any of the above therapies. NAC causes a decrease in ROS and thereby reduces hepatotoxicity by limiting systemic and local inflammation [11]. Hepatocyte uptake inhibition of amatoxins is done by mediating the OATP-1B3 transporter (OATP, organic anion transporting polypeptide) with Penicillin G and silibinin [19]. Various studies have used NAC in treating Amanita phalloides-induced liver injury at various doses. In their first series in 1992, Locatelli et al. used NAC infusion in two stages - the first one was a prolonged infusion of 150 mg/kg bolus, which was then followed by 50 mg/kg every four hours for 3 to 18 days. This study had a mortality rate of 8%, irrespective of liver transplantation [20].

The same group, in 1993, carried out the first big cohort study of amanita-induced acute liver failure patients. This time, they used the same previous loading dose of NAC 150 mg/kg intravenous. However, it was followed by 300 mg/kg/day until 48 hours post poisoning, as well as used forced diuresis until urinary amanitin levels came negative. This time, their study showed a lower mortality of 2.5% and non-worsening of liver functional tests [20]. Ahishali et al., in their case series of 77 patients, used a three times a day regimen of 70 mg/kg NAC for the first 3-5 days, on top of digestive decontamination, penicillin G and silybin, and hemofiltration. This case series reported low mortality and improving liver functional tests (LFTs) [21]. A review done retrospectively over 10 years by the California Poison Control System database found NAC to be the only treatment option leading to decreased mortality. However, treatment bias was acknowledged in the sickest of patients only [22]. A recent systematic review has shown promising higher survival rates when NAC was added to silybin compared to supportive treatment only [23]. Recent research in 2023 has given hope for developing a specific antidote against α-amanitin toxicity called indocyanine green (ICG). A catalytic enzyme for N-glucan biosynthesis, STT3B, is the necessary component in producing amanitin toxicity, and its inhibitor, indocyanine green (ICG), serves as a specific antidote [24]. This is the first time that a specific novel antidote might be used both in cells and animals to terminate AMA toxicity. This has also revealed that genome-wide CRISPR screening added with silico drug prediction can actually deliver potent antidotes to many human poisons.

Patient counseling and a good understanding of the type of edible mushroom species are very important in avoiding future mishaps.

## Conclusions

This case serves as a stark reminder of the severe hepatic dysfunction and coagulation abnormalities that can result from mushroom poisoning, especially in cases of delayed presentation. The patient's critical state upon admission underscores the life-threatening nature of this condition. However, the significant improvements seen in liver and renal function during her stay in the hospital, and the eventual discharge of the patient with significant symptomatic relief and stable hemodynamics, highlight the potential for recovery with the institution of appropriate therapeutic measures. This case underscores the crucial importance of early diagnosis and management in suspected cases of mushroom toxicity, and the potential for hopeful and optimistic outcomes with the right treatment.
